# Current effect of COVID-19 global pandemic on the professional and life profiles of the Egyptian spine surgeons

**DOI:** 10.1051/sicotj/2020029

**Published:** 2020-08-20

**Authors:** Mohamed Fawzy Khattab, Ali Mohamed Abou-Madawi

**Affiliations:** 1 Orthopedic Department, Ain Shams University Hospital 11588 Cairo Egypt; 2 Neurosurgery Department, Suez Canal University Hospital 41522 Ismailia Egypt

**Keywords:** COVID-19, Spine surgeon, Egypt, Socioeconomic

## Abstract

*Introduction*: During the recent decade, many outbreaks of infectious diseases have been reported at increasing scales and frequency. The novel COVID-19 is the most recent lethal virus and has been declared to be a pandemic disease on March 11th, 2020. It has spread from China to most of the countries around the world causing a great burden on individuals and communities. The socioeconomic and professional profiles have been affected seriously by this pandemic. The aim of this study was to assess the short-term effects of COVID-19 on the socioeconomic profile of spinal surgeons in Egypt. *Methods*: We conducted a cross-sectional online survey study to address the effect of COVID-19 global pandemic on spine surgeons in Egypt, discussing the short-term socioeconomic effect of COVID-19 global pandemic on the professional and social profiles of the Egyptian spine surgeons. A SurveyMonkey^®^ questionnaire was sent to 190 spine surgeons registered in the Egyptian spine association database. *Results*: Ninety male surgeons responded to our four-day survey. The responders included the following: 4 residents, 16 fellows, and 70 consultants working in different Egyptian hospitals. The partial country lockdown was associated with drop in monthly income and in number of both elective and emergency operations. Most surgeons either stopped surgery or limited the number of either elective or emergency surgeries as well as outpatient clinics. Most of them were not in the COVID-19 team or did not receive any training, working under immense physical and psychological stress of being exposed to transmission of infection. *Discussion*: COVID-19 global pandemic negatively affected spine surgeons in Egypt socioeconomically. The Health Authority and the community have to work jointly to help the health care professionals in overcoming this crisis.

## Introduction

Through the recent decade, many outbreaks of infectious diseases have been reported at an increasing scale and frequency. The outbreaks of these diseases include the following: in February 2003, SARS-CoV (Severe Acute Respiratory Syndrome); in September 2012, MERS-CoV (Middle East Respiratory Syndrome); more recently, in December 2019, the emergence of the novel coronavirus (COVID-19) epidemic, threatening the Chinese health care system and later on the whole world. In China, they attempted to contain the spread of the infection, thinking it to be a zoonotic infectious disease. By the end of December, in Wuhan, China, a cluster outbreak was reported and was linked to seafood and wholesale wet market [1]. Although, now, the disease was controlled in China, it has spread globally across all continents. On March 11th, 2020, the World Health Organization (WHO) declared this COVID-19 outbreak a global health emergency, calling for global solidarity and international effort to control this epidemic [2]. The patients’ population is steadily growing through different countries across the globe with variation in number in each country. While preparing this manuscript, the total number of patients was 5 488 825 with 349 095 deaths [3].

The medical staffs are working on the front lines guarding the society during this critical medical circumstance. Working under these stressful conditions, the spine surgeons who are fighting these pandemics would be greatly influenced. As a part of the medical team, spine surgeons are facing patients in outpatient clinics, inpatients wards, causality departments, intensive care units, and operating theaters. Although those patients may be asymptomatic and look fine, they might be carrying the COVID-19 disease, posing a real risk to spine surgeons, their families, neighbours, and other patients. About 80% of COVID-19 patients either have mild symptoms or are asymptomatic, so there are false negative tests [4]. Spending considerable time in operating theaters which are typically COVID-19 wind tunnels is a major risk for all attendants. So, we have to consider every patient we see or operate on as being COVID-19-positive unless proved otherwise.

In different spinal procedures, we use, drills, hammers, burrs, osteotomes, etc.; these can splash virus onto the ceiling, other metal, and surrounding surfaces. COVID-19 virus can withstand living on these surfaces for 2–3 days. Therefore, any surgery during the following 2 days will expose again all attendants to the virus sucked by the fans and blown again through the AC or ventilator system. The virus load here is very severe compared to touching an ordinary button used in our daily lives and then touching your eyes or nose [5]. This load could be deadly due to cytokine storm, causing inflammation and wreaking havoc in the lungs with subsequent long-term lung damage and fibrosis, causing functional disability [6–9]. This is why the death rate is 10–12.5% among doctors regardless of their age group. Age, underlying disease, secondary infection, and elevated inflammatory indicators in blood are predictors of fatal outcome. Mortality might be due to virus-activated cytokine storm syndrome or fulminant myocarditis [10]. Up until now, despite the ongoing investigation [11], the mainstay of clinical management is symptomatic treatment with organ support in intensive care for severely ill patients.

According to the Egyptian Spine Association Registry, we have 250 active spine surgeons. Those Egyptian spine surgeons have unique professional life due to multifactorial background, including social, economic, and scientific factors. Egypt is a unique country having many well-educated, talented, and skilled healthcare providers. The low monthly income is the drive pushing spine surgeons to work in many places including university hospitals, Ministry of Health (governmental) hospitals, military hospitals, private hospitals, and their own private outpatient clinics. *The COVID-19 pandemic resulted in a wide range of socioeconomic consequences including disruption of trade and travel, psychological and physical burden, severe pressure on the medical health service and suppliers, and loss of lives and sickness* [12]. The aim of this study is to assess the short-term effects of COVID-19 on the socioeconomic profile of spine surgeons in Egypt.

## Material and methods

Cross-sectional online survey study to address the effect of COVID-19 global pandemic on the life and professional profile of spine surgeons in Egypt was conducted. This survey aimed to address the short-term socioeconomic effect of COVID-19 global pandemic on the professional and social profiles of the Egyptian spine surgeons. To our knowledge this was the first study of the short-term socioeconomic effect of COVID-19 novel virus on Egyptian spine surgeons. The questions in the survey were designed by the authors and not quoted from any other platform due to the unique characteristics of the Egyptian spine surgeons. The questionnaire was divided into 10 clear, simple, and reproducible questions, each one meant to measure specific parameter. The main parameters were economic factors, practice, knowledge, and attitude towards the COVID-19 novel virus.

During the period between March 30th and April 2nd, 2020, during the country’s lockdown period, a SurveyMonkey^®^ questionnaire was sent to 190 spine surgeons registered in the Egyptian spine association database. The questionnaire consisted of 10 questions: (1) assessing the professional rank; (2) evaluating place of work; (3) analyzing the effect on monthly income; (4) identifying the drop in the number of outpatient clinic cases; (5) identifying the effect on emergency spine cases; (6) assessing if there is postoperative complications, especially the respiratory complications; (7) examining the surgeon’s response to the lockdown due to the pandemic; (8) seeing if any spine surgeons participate in COVID-19 team; (9) seeing if any spine surgeons got training to deal with COVID-19 pandemic; (10) assessing the surgeon’s health state in relation to the current pandemic.

### Statistical analysis

In this short-term descriptive study, a descriptive statistic was performed using the mean and standard deviation for numerical data while number and percentage were used for qualitative data. Analysis was performed by Statistical Package for Social Science SPSS (version 20, Chicago, Inc.).

## Results

Descriptive cross-sectional survey study has been conducted to assess the burden of the novel coronavirus global pandemic on Egyptian spine surgeons. A total of 90 Egyptian spine surgeons out of 190 were able to complete the questionnaire at the given week from March 30th, 2020, to April 2nd, 2020. The survey responders were as follows: 35 responded on March 30th; 16 on March 31st; 36 on April 1st; and the last 3 on April 2nd.

The responders were 4 (4.4%) orthopedic residents, 16 (17.78%) spine fellows, and 70 (77.78%) spine consultants. All were males and representing the different sectors of spine health care system in Egypt. The Egyptian spine system allows doctors to work in many hospitals. This survey showed that 37 (41.11%) spine surgeons were working in university hospitals, 10 (11.11%) in the Ministry of Health, 9 (10%) in private hospitals, and 5 (5.6%) in private offices, and 29 (32.22%) spine surgeons were working in more than one health care providing hospitals, including university, Ministry of Health, private hospitals, and private offices (Table 1). From the above described criteria of the 90 responders, we consider that those 90 surgeons represent and speak for the whole spine surgeons in Egypt.

**Table 1:** Distribution of spine surgeons according to workplace.

Workplace	Responders (%)
University hospitals	37 (41.1)
Ministry of Health hospitals	10 (11.1)
Private hospitals	9 (10)
Private office	5 (5.6)
All of the above	29 (32.2)

The direct economic effect on the spine surgeon’s monthly income was addressed by the questionnaire, and it was shown that 78 (86.7%) spine surgeons experienced a drop in their monthly salary and 12 were not affected. Overall, 37 (41.11%) spine surgeons lost 50% of their monthly income, 22 (24.44%) lost 80%, 12 (13.33%) lost 70%, and 6 (6.67%) lost 20%; 12 (13.3%) have the same income, and one resident who is working in a private hospital lost all of his monthly income due to lockdown (Table 2).

**Table 2:** Effects of COVID-19 on spine surgeons’ monthly income.

Income loss (%)	Responders (%)
0	12 (13.3)
20	6 (6.7)
50	37 (41.1)
70	12 (13.3)
80	22 (24.4)
100	1 (1.1)

During this lockdown and fear of catching COVID-19 infection, 24 (27%) spine surgeons stopped the outpatient clinics completely, but 73% are still running it with a drop in the number of cases: 42 (46.67%) spine surgeons reported a drop of 75% of their outpatient clinic cases, 19 (21.11%) a drop of 50%, and 5 (5.6%) a drop of 25% (Table 3).

**Table 3:** Effect of COVID-19 on outpatient clinics of spine surgeons.

Outpatient clinic performance	Responders (%)
Stopped outpatient clinic	24 (26.7)
Running clinic with 75% drop	42 (46.7)
Running clinic with 50% drop	19 (21.1)
Running clinic with 25% drop	5 (5.6)

**Figure 1:**
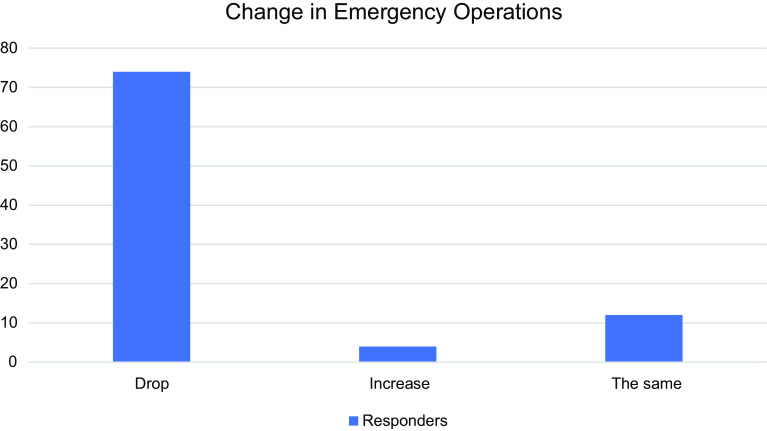
Effect of COVID-19 on the rate of emergency spine cases.

Egypt suffers from heavy traffic congestion and spine trauma is a common practice among spine surgeons. During this global pandemic, 74 (82.2%) spine surgeons reported that the number of spine emergency cases dropped down, 4 (4.4%) reported an increase in the number of emergency cases, and 12 (13.3%) reported the same rate as before the pandemic (Figure 1).

**Figure 2:**
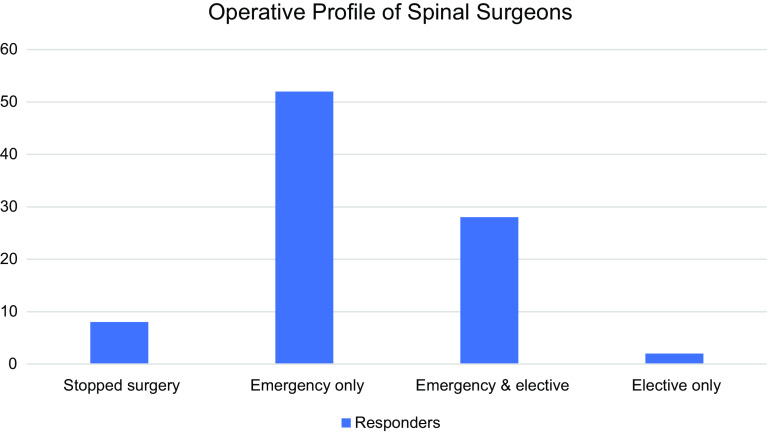
Effect of COVID-19 on profile of surgical practice among spine surgeons.

In order to avoid depletion of our healthcare system resources, the governmental hospitals either stopped or decreased the number of the elective cases, while the private hospitals continued operating elective cases. This study survey addressed the surgeon’s response and it reported that 8 (8.9%) spine surgeons stopped operation at all, and 82 were still operating. Those 82 operating surgeons included 52 (57.8%) surgeons operating only on emergency cases; 28 (31.1%) spine surgeons, both elective and emergency cases; and 2 (2.2%) surgeons, only elective cases (Figure 2).

Operating in such a pandemic is risky for the surgeon and the patients as well, and the incidence of complications may increase, especially the postoperative chest complications. The postoperative period for 95% of the operating spine surgeons’ patients was uneventful, while 5% of surgeons noticed postoperative respiratory problems with their cases including one case of pneumonia. Laboratory tests were negative for COVID-19. This case went under operation in a university hospital as an emergency case and interestingly this surgeon did not receive training on how to manage COVID-19 pandemic. Another surgeon was confronted with a case of postoperative respiratory complication suspected to be COVID-19 infection, and later on, it turned out to be COVID-19 negative. He is a consultant working in a university hospital, his monthly income dropped by 80%, he did not receive training on how to deal with COVID-19 pandemic, and he stopped surgeries after that event.

Some hospitals asked spine surgeons to help in leading or assisting COVID-19 team. This survey study reported that 95% of the spine surgeons did not participate in COVID-19 team, while only 5 spine surgeons (5.6%) participated COVID-19 team. Asking the surgeons if they had got training for this COVID-19 global pandemic, we found that 23 (25.6%) had training, while 67 (74.4%) spine surgeons did not receive training on how to manage or deal with COVID-19 patients.

Health care providers may be exposed to asymptomatic COVID-19 cases. That is why asking the spine surgeons about their health state in the current pandemic is essential. In this study, 11 spine surgeons had some symptoms related to the disease such as sore throat or cough; however, none of them have been tested for COVID-19 or proved to have it. The other 79 spine surgeons were asymptomatic and healthy.

## Discussion

The current COVID-19 global pandemic has global health and socioeconomic burdens. Spine health care providers and the whole community may be drastically affected. Egypt is an important country in the middle east region that has a unique health care system. Once COVID-19 cases started to be diagnosed and reported, the government started to implement mitigation policy. By the night of 25 March, community lockdown has been applied from 7 pm to 6 am and Friday and Saturday were assigned as a complete lockdown. This has a major effect on the community, health care providers, and spine surgeons [13].

The COVID-19 global pandemic crisis negatively affects the professional and life profiles of the Egyptian spine surgeons in many aspects. These aspects include the following: (1) rules directed by the government such as lockdown to decrease the number of COVID-19 infected cases; (2) fear of patients to go outside and seek spine surgeons’ advice; (3) risk of infection for doctors as they visit hospitals; (4) fear of transmitting infection to their family and patients; (5) fear of getting more postoperative complications, especially the postoperative respiratory infection; (6) some reports concluding that the smoke resulting from using Bovie electrocautery in spine wound exposure can negatively affect respiratory system; (7) some reports highlighting that the high-speed burr usage can lead to spread of COVID-19 virus from symptomatic patients; (8) possible aerosolization of the virus from asymptomatic patient during intubation or extubation being a risk, in a negative flow operating theaters, as the virus might settle in the wind tunnel; (9) limited availability of Personal Protective Equipment (PPE).

Cross-sectional survey study was conducted addressing the short-term effect on spine surgeons; 90 out of 190 spine surgeons registered in the Egyptian spine association have responded to the survey. Some of the registered Egyptian spine surgeons work in the Gulf region or in different western countries and this explains the difference between survey responders and the registered surgeons. 17.78% of the responders were fellows and 4.44% were residents; this demonstrates that most of the residents and young fellows are busy in the hospital’s emergency rooms.

Egyptian spine surgeons have limited monthly income; for that reason, most of them work in many places; the availability of private hospitals and national geographical distribution may affect this rule. This study showed that 32.2% are working in more than two hospitals and private offices; this may reflect the stressful daily life, and the time spent in driving between these hospitals which should be considered as stress time as well. This may add risk of harvesting infection from different sources.

The monthly income was lost totally for one resident working in private hospital. The younger the surgeon is, the more vulnerable he is to economic crisis. The monthly income was not affected in 13% of cases, as they are consultants working in many hospitals and have private offices. From this survey, if the lockdown is implemented for a longer period and the physician is young and is working in private hospital, he is more vulnerable to economic drawbacks. The medical syndicate must be aware of that and offer support to the young generations during these hard times.

The number of spine outpatient clinic visitors dropped. The prime minister announced partial country lockdown from 7 pm to 6 am, plus complete country lockdown is implemented on Friday and Saturday. In this survey, 27% of outpatient clinics were stopped; the presence of health care providers is important, and some colleagues joined the telemedicine as a backup; it is free to help patients to stay at home and seek free medical advice online. The drop in the emergency cases is due to country lockdown and decrease of pedestrians and car accidents, which may decrease the number of trauma cases.

According to the authority recommendations during pandemics, it is important to maintain all the health care system resources and to reduce their depletion. It was recommended to decrease the number of elective surgeries to decrease the burden on intensive care units and reserve ventilators for needs. The response varies from private to governmental hospitals; Does the economic factor have a role? maybe! New guideline has been issued from the NASS to assist spine care providers and health care authorities in making informed decisions on behalf of spinal disorders patients during this difficult time [14]. Decision-making should take into consideration the following: (1) local conditions, policies, rules, and regulations; (2) patient conditions, including health status, risk of illness, and risk of exposure to COVID-19; (3) staff availability and PPE considering preserving resources; (4) current and projected local COVID-19 cases [14]. We followed these recommendations and other ones as well [15], as it is the necessity and current status that we have to deal with.

Despite this, a minority of colleagues still operate the elective cases; the lockdown, the economic state, and the need to have salary are the driving forces. This issue has been reported by Ghogawala et al. [16] as a consequence of a reduction in elective surgery through which revenue is lost by hospitals and surgeons, and the magnitudes of the effect of revenues lost on surgeons vary widely depending on the institutional model for compensation.

It is important during the pandemic to decrease the surgeries to limit the burden on health care system; respiratory complication may be anticipated especially in emergency cases, anticipated more in university hospitals, and we noticed that surgeons are not trained to deal with the COVID-19 pandemic.

It is of utmost importance and its one of the rules by the local health administration to supply all the medical health care and spine surgeons with the up-to-date Personal Protective Equipment (PPE). We have limited resources in our country and we use only conventional surgical masks and gloves as PPE in many centers. The psychological aspect of the spine heath care providers goes side by side with the physical aspect of this pandemic. They know well that this infection is easily transmitted and it can be lethal and, with such exposure with inadequate PPE, they work under severe pressure. There are some medical health care facilities that have been locked down due to reported COVID-19 among some of its staff. Our survey study showed that 12% of our sample have some minor symptoms such as cough or sore throat and they panicked and sought medical consultation. So far, we did not have any reports of COVID-19 among spine surgeons in our country.

No doubt that the medical health care profile, including the Egyptian spine care providers, has changed through this COVID-19 pandemic. These changes include the following: (1) decrease in the number of elective and emergency operations; (2) increase in the number of telemedicine clinic sessions; (3) increase in enthusiasm and researches; and (4) increase in the number of online training and educational webinars. This change in practice profile of Egyptian spine surgeons has been recently reported by Indian study from Ophthalmological group who reported similar results [17]. They reported that majority of Indian Ophthalmologists stopped seeing patients during the lockdown with near total cessation of elective surgeries, while the emergency services were still being attended to by 27.5% of the responders. Another study [18] from Marseille, France reporting the possibility to take care of spinal patients during the COVID-19 pandemic. They reported a decrease of 50% of total number of surgical procedures. This included a stable number of tumors and infection while a significant decrease in the number of trauma and degenerative cases. To the best of our knowledge there is no report of the effects of COVID-19 novel virus on socioeconomic profile of spine surgeons. Most of these reports were concerned with personal protection and updated guidelines for what is urgent or emergent during the pandemic lockdown [14, 19, 20].

### Study limitations

The number of participants of spine surgeons in the survey undertaken was limited, although they represent whole community of spine surgeons. There is lack on data of the emerging disease’s pathogenesis, fatalities, natural history, and management.

## Conclusion

COVID-19 global pandemic negatively affected spine surgeons in Egypt socioeconomically. The Health Authority and the community have to work jointly to help the health care professionals in overcoming this crisis.

## Disclosures of Conflict of Interest

The article does not contain information about medical device(s)/drug(s).

No funds were received in support of this work.

The authors do not report any conflict of interest, any research funding, nor any sort of support, whether financial, personal payment, or any other benefit from a commercial entity.
